# Thermal Mechanical Properties of Graphene Nano-Composites with Kevlar-Nomex Copolymer: A Comparison of the Physical and Chemical Interactions

**DOI:** 10.3390/polym12112740

**Published:** 2020-11-19

**Authors:** Jessy Shiju, Fakhreia Al-Sagheer, Zahoor Ahmad

**Affiliations:** Department of Chemistry, Faculty of Science, Kuwait University, Post Box 5969, Safat-13060, Kuwait; jessyshiju@ku.edu.kw (J.S.); Falsagheer@ku.edu.kw (F.A.-S.)

**Keywords:** aramids, graphene, nano-composites, interfacial chemical bonding, visco-elastic properties

## Abstract

This paper reports the preparation of Kevlar-Nomex copolymer nano-composites with exfoliated pristine and functionalized graphene sheets (Grs). The graphene oxide (GrO) platelets were amidized by the reaction of amine-terminated aramid (Ar) with the functional groups present on the GrO surface to prepare the nano-composites films with different loadings of GrO. Chemical changes involved during the oxidation and subsequent amidation were monitored by Raman, FTIR and XP spectroscopic analyses. Morphology of the composite films was studied by atomic force and scanning electron microscopies. Viscoelastic properties of the hybrid films were studied for their glass transition temperature (Tg) and storage modulus by dynamical mechanical thermal analysis (DMTA). A higher shift in glass transition temperature was obtained by chemically binding the aramid copolymer chains on the functionalized Gr sheets. The increase in tensile strength and modulus at various loadings of GrO are compared with the composites using pristine Gr. The effect of interfacial interactions between the matrix chains and the reinforcement on the properties of these hybrids have been explained.

## 1. Introduction

High polymers, because of their relatively low density, cost, ease in processability and high chemical resistance, are considered an important class of materials used in modern technology, but as structural materials, they have limitations due to their low modulus, mechanical strength and low operating temperatures. Reinforcement of polymers with fibers or particulate fillers permits the fabrication of composites [1,2,3], which can overcome some of these shortcomings. Polymeric composites are characterized by their high specific strength, stiffness and impact resistance and have become very popular over the last three decades. The reinforcing effect of the fillers in these depends on many factors [4] such as the type of fillers, shape, size and the aspect ratio. The reduced size of the fillers offer many advantages [5,6], such that the low contents of nano-fillers can provide better improvement in mechanical properties without affecting their processing ability. The full potential of property enhancement in polymers, however, can be gained by their uniform dispersion states, which are necessary to provides large surface interaction between the filler and the matrix.

Carbon-based nano-fillers such as carbon black, carbon nano-tubes (CNTs) and carbon nano-fibers (CNF) have attracted a lot of attention in recent times and have been used extensively [7,8,9,10] for the preparation of polymer nano-composites. Among these, very effective fillers that enhance the mechanical and conductive properties in polymers are CNTs [10,11,12,13]. But the higher cost of production of CNTs is a major drawback that can hinder industrial scale production of such composites [14]. A promising filler to replace CNTs for polymer composites is graphene (Gr), which has a two dimensional sp^2^-hybridized carbon platelet structure. These sheets, with one-atom thickness, have gained importance over the last two decades [15,16,17,18]. Compared to CNTs, the Grs are much cheaper, because these can be easily produced on a mass scale from a graphite precursor. Some of the many important characteristics, such as high mechanical strength, outstanding electric and thermal conductivity, low coefficient of thermal expansion, high aspect ratio and flexibility make Gr an extraordinary reinforcing filler for polymeric materials [19,20,21,22]. Although CNTs can provide a network structure in the matrix to improve electrical and thermal transport, the inner tube surface is not accessible to polymer chains and this makes Gr a preferred filler [23] over the CNTs. Based on the literature available on the fabrication, properties, and polymer composites, Gr has proved to be a promising filler [24,25] with potential applications in the field of electronics [26,27], sensors [28] energy production and storage [29,30] and bioengineering [31].

The improvement in the mechanical, electrical, and thermal properties of polymer physicochemical properties of the nano-composites mainly depends [32,33] on the distribution of Gr layers in the polymer matrix. A homogeneous and uniform dispersion of Gr in the matrix is required so that the external load may be efficiently transferred under the stress condition through strong interfacial interactions between Gr layers and the polymer matrices. The inherent nature of Gr, however, makes its dispersion difficult within the majority of polymers. The most common route, therefore, is to use oxidized graphene (GrO) sheets followed by reduction and mechanical exfoliation processes [33,34,35]. The basis of oxidation chemistry for the production of graphene oxide (GrO) is the Hummers and Offeman’s processes [34,36]. The GrO sheets bearing hydroxyl and carboxyl functional groups with some epoxide, diols and ketones groups located at the edge of the sheets can help to develop van der Waals interactions with the matrix. The GrO is nonconductive, but due to its hydrophilic nature, it can readily swell and disperse in the matrix. Recently, some new methods of Gr functionalization [37,38,39,40,41] have also been reported in the literature, which demonstrates an improved dispersibility in organic solvents and polymers. The wrinkled nature of oxidized Gr plates with a high surface can provide stronger interfacial interaction with the matrix [23]. The interaction of GrO with the polymer matrix leading to exfoliation of the nano-sheets have been found to improve significantly the physical, mechanical, thermal, electrical and electronic properties of the matrix materials.

Three important processing techniques that have been used [38] to disperse Grs in the polymeric matrices include solution mixing, melt blending and in-situ polymerization. Among the spinning methods, wet- and melt-spinning have also been tried [41]. The solvent mixing may involve mechanical mixing, magnetic agitation or high energy sonication to give a uniform Gr dispersion. The composites can then be obtained by the solvent elution techniques. Different polymeric systems, i.e., polyurethanes [42], polypropylene [43] epoxies [44], poly(methylmathacrylate) [45,46] polyimides [47] and others have been prepared with Grs using this technique. In-situ polymerization is another technique where GrO is mixed with monomers or pre-polymers to obtain GrO-polymer composites. Polymerizing the monomers or pre-polymers with GrO gives a uniform dispersion and improved properties. The in-situ polymerization of epoxies [48,49], PU [50], PMMA [51] Polystyrene [52] and polyamides [53] with Grs have been reported in the recent past. Viscosity increase accompanying the polymerization process, however, can hinder the dispersion in this method, whereas re-stacking of graphene sheets can also significantly reduce the effectiveness of the filler.

The non-covalent bonding can also be developed with the pristine Gr with such polymers that have extended aromatic rings on the chain, i.e., aromatic polyamides, as these have highly mobile pi electrons (π and π*) located on their chains. The multiple π–π interactions between the exfoliated monolayer’s crystalline structure of Gr and aramids can provide improvement in the mechanical properties [54,55]. An important advantage of non-covalent bonding is that for most applications the Gr lattice does not get disrupted and the properties such as thermal or electric conductivity and mechanical strength are, therefore, not affected.

For improved strength and conductance, the aromatic polyamides having phenyl groups can be considered as the best candidates as a matrix due the advantage of π–π stacking with Gr fillers [55]. Fan et al. [56] have recently used a functionalized aramid nanofiber (ANF) by dispersing graphene in the dimethylsulfoxide solution of ANF. The ANFs got absorbed on the Gr nano-sheets and could be easily exfoliated and dispersed in the solvent. A combination of these materials, i.e., ANFs with graphene sheets, was used as a nano-reinforcement in the PMMA matrix, thus improving its modulus and tensile strength. Dopamine modified aramid fibers have also been grafted with amine-functionalized graphene to improve the interfacial adhesive performance [57].

Enhanced mechanical properties of aromatic polyamide fiber or films can be very useful as ultra-strong membranes, coatings and for many other advanced applications [58]. In the present work we have used a high molecular weight copolymer matrix from Kevlar^®^ and Nomex^®^-derived chains [59]. These are considered to be high performance polymers but both as such are insoluble in the organic solvents. The backbone structure of aromatic polyamide chains was suitably modified by introducing a few kinks in the linear Kevlar intractable chain. The purpose was to improve its solubility and hence processability, without affecting its outstanding thermo-mechanical performance. In the linear poly(p-phenylene-terephthalamide) Kevlar^®^ chain, nearly 30% of meta-linkages were introduced. The resin thus prepared was reinforced with exfoliated pristine Gr and GrO sheets. The carboxyl groups on the surface of GrO were chemically bonded with the amine-terminated high molecular weight aramid copolymer chains. The composite films using different loadings of functionalized GrO were prepared and the properties of these are compared with similar composites using pristine exfoliated Grs. These properties are then related with the composition and different types of possible interactions, i.e., physical or chemical, between the Gr sheets and the aramid matrix.

## 2. Experimental

### 2.1. Materials

The monomers 1,4 phenylenediamine and 1,3 phenylenediamine, terephthaloyl chloride (TPC) (99%) and the solvent dimethylacetamide (DMAC) all were obtained from Sigma Aldrich (St. Louis, MO, USA) as Analytical grade reagents. Pristine single layer Gr (99.3% purity, Thickness: 0.55–1.2 nm, Diameter: 1.5–2 μm) and single layer GrO (99.3% purity, Thickness: 0.43–1.23 nm, Diameter: 1.5–5.5 μm) were obtained from the US research nano materials. All other chemicals and reagents were used as received.

### 2.2. Synthesis of Aramid Matrix

A mixture of 1,4- and 1,3-phenylenediamine, (0.025 mol) in molar ratio 35:65 respectively, was placed in a 250 mL conical flask. To this mixture, 100 g of DMAC was added and then stirred for 30 min for complete mixing. To this solution, 0.025 mol of TPC was added under complete anhydrous conditions. The reaction mixture was stirred continually for 24 h after which the polymerization reaction was assumed to be complete. This resin was then used as a stock solution to prepare the composite films with different loadings of pristine Gr.

### 2.3. Preparation of Aramid Graphene (ArGr) Composite Films

**Scheme 1:**
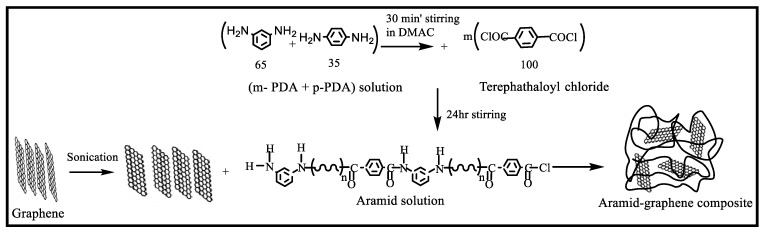
Preparation of ArGr Composites.

Pristine Gr (0.300 g) was added in 15 g DMAC and sonicated in a 100 mL flask for 2 h and then allowed to stir for another 24 h to separate the sheets. Different amounts of pristine Gr solution were added in a given amount of aramid solution prepared in Section 2.2. The films with 0, 2, 4, 6 and 8 wt % of Gr were prepared with constant stirring for 24 h to achieve the maximum dispersion. Thin films with uniform thickness were cast at 70 °C using a solvent elution technique. In order to remove the HCl produced during the polymerization reaction, these were washed repeatedly with distilled water. The films were then dried for 72 h at 80 °C and then at 120 °C under vacuum for 24 h for complete removal of the solvent (Scheme 1). These composite films are referred to as Ar-Gr films in this paper.

### 2.4. Preparation of Aramid Graphene Oxide (ArGrO) Composite Films

**Scheme 2:**
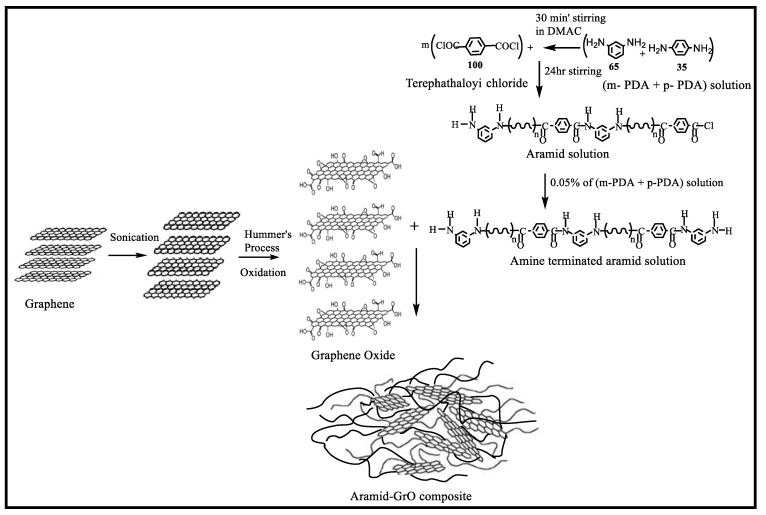
Preparation of ArGrO Composites.

The aramid resin was prepared as described in Section 2.2. To this resin at the end of the polymerization reaction, an additional amount, i.e., 0.05 mol % of the initial amount of a mixture of p-and m-phenylendiamines, was added followed by 3 h of stirring. An amine-capped aramid chain matrix was then used as a stock solution for the preparation of AR-GrO composites (Scheme 2).

The GrO (0.3 g) was taken in 15 g DMAC and the dissolution was obtained by 2 h sonification with a further stirring for 24 h. Different amounts of GrO in solution were added to a given amount of aramid solution and the mixture was then heated for 3 h at 60 °C to react the amine end groups of the polymer chain with the –COOH groups present on the surface of GrO. The mixture was further stirred for 24 h at an ambient temperature. ArGrO composite films with 0, 2, 4, 6 and 8 Wt. % of GrO were thus prepared as described in the Section 2.3

### 2.5. Characterization of the Composite Films

The interaction of Grs with the polymer matrix, their degree of dispersion, morphology and the thermal mechanical behavior of the composite films were studied using a variety of techniques as given below.

#### 2.5.1. Fourier Transformed Infrared (FTIR)

Absorption spectra for the pristine and oxidized Grs were taken using FTIR Spectrometer JASCO-6300 in the range of 400–4000 cm^−1^ on the KBr disk at a resolution of 4 cm^−1^ to confirm the structural differences between the two types of Grs.

#### 2.5.2. Raman Spectroscopy (RS)

Raman Spectroscopy was carried out on Renishaw inVia Raman Microscope using a 514 nm Laser under objective lens with ×100 magnification giving exposure time of 10 s. Area under the D and G bands was measured and their ratio (I_D_/I_G_) was used as a measure of de-bundling of platelets due to functionalization carried out in the regular structures of Gr.

#### 2.5.3. The X-Ray Photoelectron Spectroscopy (XPS)

High-resolution XPS spectra was taken to investigate the chemical reaction between the acid-functionalized (GrO) and amine-terminated aramid chains. The elemental composition of N, C and O functionalities at the sample surface were analyzed. Thermo ESCALAB 250 Xi was used with a monochromatic A1Kα radiation source (1486.6 eV). The degassed samples were kept for 12 h within the XPS chamber before analysis. Thermo Advantage software Version-4.87 was used for the spectrum acquisition.

#### 2.5.4. Atomic Force Microscopy (AFM)

The AFM images of composite films (ArGr and ArGrO) were obtained by Nano-scope IV Scanning Probe Microscope (Santa Barbara, CA, USA) using contact AFM in a constant force mode (1 ± 2 nN) under air. The cantilever was gold coated silicon nitride with a silicon tip of 20 nm radius. The images were acquired under tapping mode “RTESP” tips. The backgrounds from the images were subtracted using nano-scope software.

#### 2.5.5. Scanning Electron Microscopy (SEM)

Morphology of aramid composites with both types of Grs was studied by SEM user Interface Model SUPRA-50 VP (JDISS). (Oberkochen, Germany). The films were fractured using the microtome at −20 °C and coated with platinum. The fractured films were examined at 15 KV.

#### 2.5.6. Dynamic Mechanical Analysis (DMA)

A Dynamic Mechanical Analyzer Q-800 from TA (New Castle, DE, USA) was used to measure the viscoelastic behavior of the films. The measurements were taken under a tension mode in the temperature range of 50–500 °C. The heating rate was kept at 5 min/°C using frequency 5 Hz under inert atmosphere with a floating pressure of 60 Pa. The glass transition temperature (Tg) involving alpha-relaxations was measured from the peaks of the curves obtained from the tan δ vs temperature plots.

#### 2.5.7. Tensile Analysis

Tensile measurements on pure aramid and both types of composite films at 25 °C were carried out on Shimadzu Autograph TRAPEZIUM X (Kyoto, Japan). The ASTM D-882 (standards) were used. The value of strain rate was 5 mm/min and a minimum of 5 samples were used for each measurement.

#### 2.5.8. Thermogravimetric Analysis (TGA)

TGA was carried out using Shimadzu TGA-50 analyzer (Kyoto, Japan) from ambient to 800 °C. The heating rate was 10 °C/min with an inert atmosphere using pure nitrogen. The first derivative of thermograms was used to measure the thermal decomposition temperature.

The property-structure relationship and its dependence on composition, interfacial interactions and the changing morphology in the resulting composites is now discussed for the designing of the new high performance nano-composites.

## 3. Results and Discussion

### 3.1. FTIR Analysis

**Figure 1:**
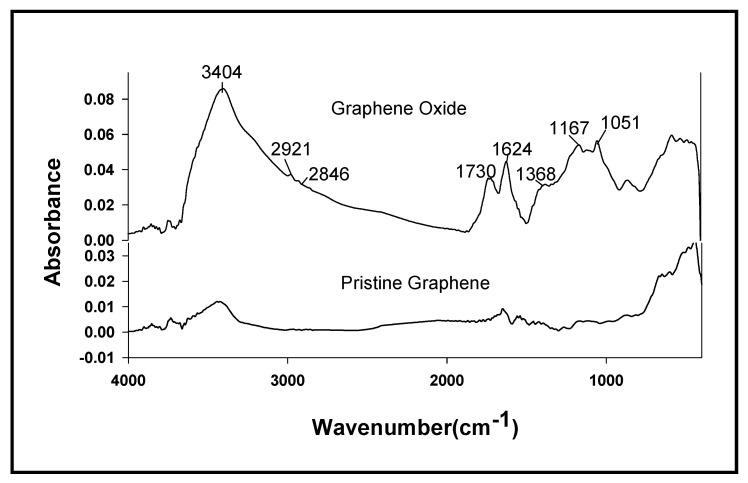
FTIR Spectra of the Pristine Gr and GrO.

Fourier Transform Infrared (FTIR) analysis on the pristine Gr and GrO was performed to confirm the possible structural differences in the samples and these spectra are given in the Figure 1. The presence of the peak at 1624 cm^−1^ for (C=C) bonds depicts the integrity of the hexagonal structure of Gr. All intensities of the peaks corresponding to the oxygen-containing functionalities are significant in GrO in comparison to the spectra obtained in pristine Gr. The presence of hydroxyl groups is seen as a broad peak observed in the range of 3404 cm^−1^, whereas the carboxyl groups are seen at 1730 cm^−1^. The carboxyl peak has shifted in fact from 1750 cm^−1^ due to the conjugation effect by the ring structure of Gr plates to a lower wavelength of 1730 cm^−1^. The peak is also overlapped near the 1630 cm^−1^ region by the hydrogen-bonded carbonyl group stretching.

Besides, two weak peaks at 1167 cm^−1^ and at 1051 cm^−1^ also appeared in GrO, which are due to the C–O stretching deformation mode of the carboxylic acid group. In the spectrum of the GrO the peak at 1368 cm^−1^ is a single bonded carboxyl (C–O)–O. Another small peak at 2921 cm^−1^ is due to the (C–H) asymmetric and symmetric stretching vibration derived from the long alkyl chain. The peaks due to the oxygen functionalities present in GrO are almost completely absent in the spectrum of Gr. The high intensity of the main peaks in GrO confirms the presence of a large amount of oxygen functional groups due to the oxidation process that are required to develop a further reaction of GrO platelets with the aramid matrix chains.

### 3.2. Raman Spectroscopy (RS)

**Figure 2:**
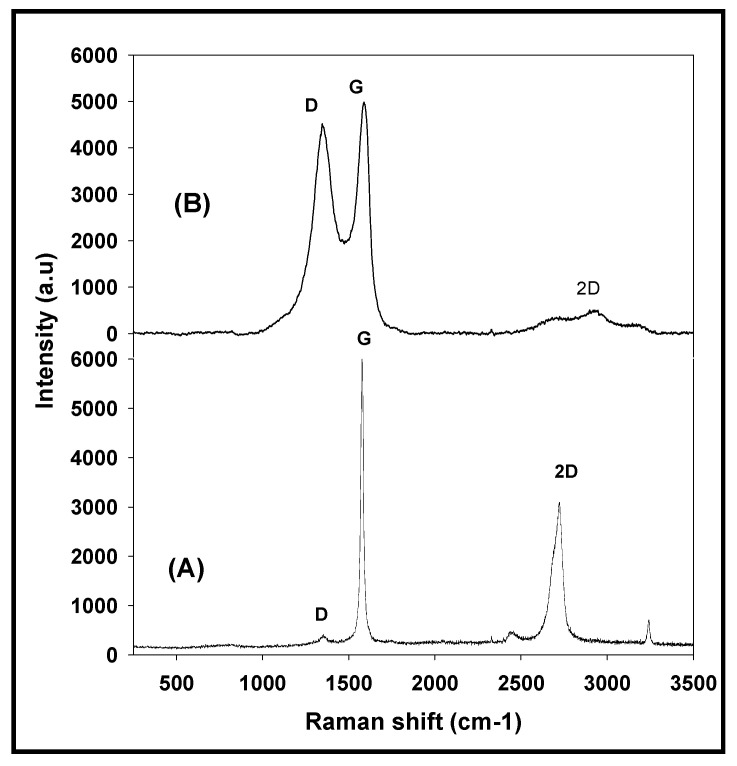
Raman spectra of pristine Gr (**A**) and GrO (**B**).

The Raman spectra for GrO and Gr platelets are shown in Figure 2. Three bands: D (1348 cm^−1^), G (1588 cm^−1^) and 2D (2680 cm^−1^) are characteristic for Gr material. The ordered high frequency in-plane stretching of the C–C bonds gives rise to a G band of around 1588 cm^−1^. The band appearing at 2680 cm^−1^ is due to the double resonance process that is observed in the defect-free SP^2^ carbons. The presence of G and 2D bands alone is the characteristic feature of pristine Gr sheets. The D band observed at 1348 cm^−1^ in GrO is due to the out-of-plane vibrations (breathing mode) attributed to the de-bundling in GrO platelets due to oxidation and it corresponds to the presence of edge carbons, defects and disordered carbons (structural defects). The intensities of D and G bands give an idea regarding the extent of dis-orderliness in the structure. The increase in I(D)/I(G) from 0.0775 to 0.7810 is attributed to a conversion from sp^2^-bonded carbon found in pristine Gr into carbon with substantial sp^3^ bonding in GrO, an indication of higher defects introduced by the presence of different functional groups produced during the oxidation process.

### 3.3. X-Ray Photoelectron Spectroscopy (XPS)

**Figure 3:**
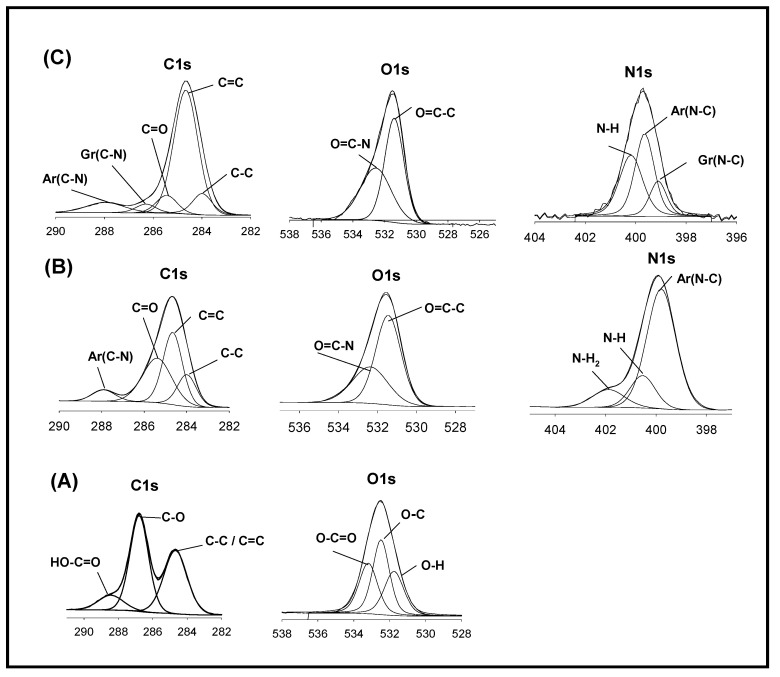
XPS spectra: GrO (**A**); Aramid pure (**B**); ArGrO Composite (**C**).

XPS, a surface sensitive technique, was used here to analyze the surface chemical composition and bonding between GrO and aramid matrix. The surface elemental composition of C, O, and N were identified for the functional groups present in the chemically bonded ArGrO composites. Figure 3 depicts the XPS spectra of GrO (A); neat polymer matrix (B) and the ArGrO (C) provide information about the bonding developed between the matrix and GrO.

XPS investigation on GrO shows the three peaks of C1s; at 284.68 eV attributed to the sp^2^ carbon bonds, 286.79 eV to C–O bonds and 288.43 eV to HO–C=O bonds, respectively [60]. The peaks of O1s spectra in GrO are assigned to O–C (532.49 eV), O=C–O (533.20 eV) and O–H (531.76 eV) groups, respectively. The relative area of the peak at 286.79 in fact increases after oxidation in comparison to that at 284.68 with pristine Gr [61,62] due to an increase in the number and contribution of Gr-derived sp^3^ carbon bond after its oxidation. It is noted that the wt. % of C=O at 288.43 also increases considerably, indicating the presence of –COOH groups on the surface of GrO [62,63]. There is no N 1s peaks observed, as expected in the GrO sample.

The XPS spectra for the aramid chains show peaks at 283.99 eV as well as 284.66 eV for C–C and C=C bonds respectively. Carbonyl peaks in the amide (–NHC=O) linkage can be seen at 285.38, whereas the (C–N)–Ar bond from the aramid chain shows its peak at 287.90 eV. The O1s peaks are assigned to O=C–C at 531.34 eV and O=C–N at 532.37 eV in the neat aramid chains. In the nitrogen spectrum, unreacted primary amines NH_2_ attached as the terminal end group of neat aramid chains are confirmed at 401.88 eV and amide N–H at 400.53 eV.

In ArGrO composite, the disappearance of carboxyl C–O from the graphene oxide and the absence of NH_2_ peak at 401.88 eV confirms the amide formation due to the reaction between NH_2_ and COOH groups and as expected, the N–H bond from the polyamide chains can be seen at 400.53 eV. The intensity of O=C–N at 532.37 eV in the composite increased due to the inclusion of GrO by the amide formation. For O1s, the peaks at 531.38 eV and 532.37 eV are assigned to O=C–C and O=C–N, respectively. There is a considerable increase in the atomic % of oxygen (the oxygen concentration) which is attached to the carbon atom bonded to aromatic carbon due to the inclusion of GrO in the matrix.

### 3.4. Atomic Force Microscopy (AFM)

**Figure 4:**
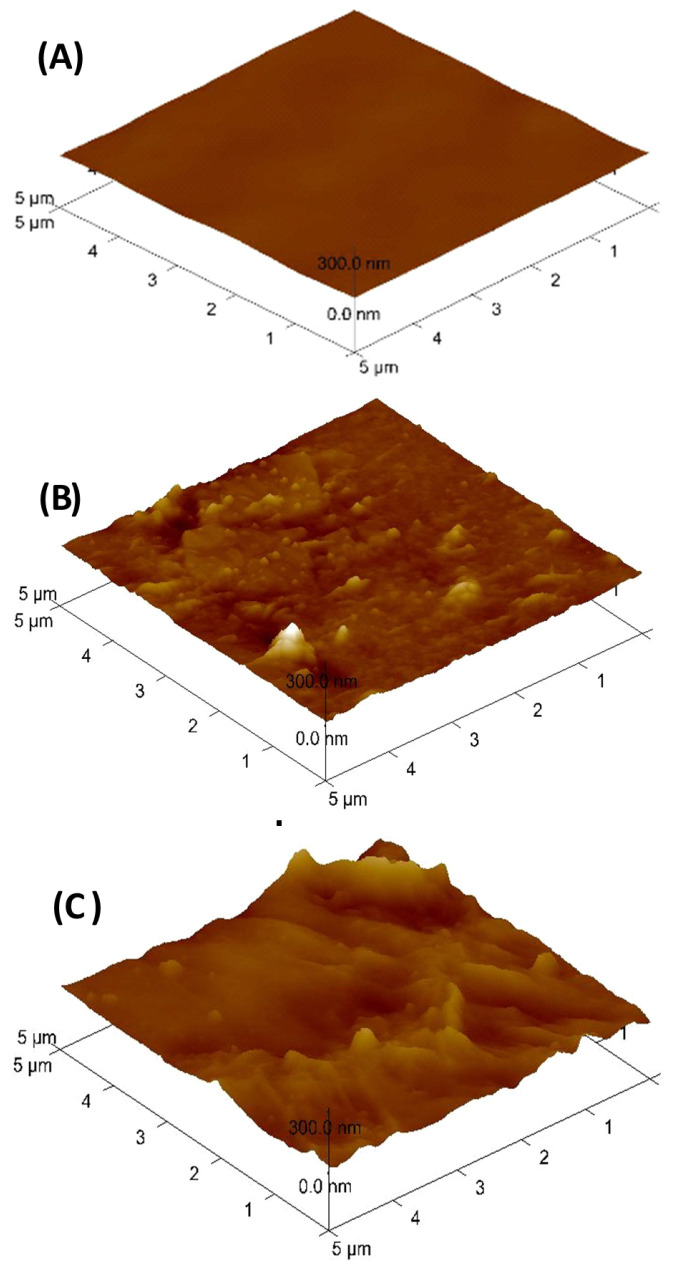
AFM images: neat aramid (**A**); ArGr composites (**B**); ArGrO composite(**C**) both with 8 wt.% Grs.

The surface topology of the ArGr and ArGrO composite films was studied by AFM to analyze the roughness and the dispersions of Gr in the matrix. Figure 4 shows the representative AFM surface topology of the samples of neat aramid matrix film and those containing 8 wt. % Grs. Surface of the pure matrix is seen as being smooth and flat, but the topology features of the composite films are dependent on the graphene loadings and their type.

The ArGr composite film depicts a mountain-valley feature with their height varying from 5 to 300 nm. The sharp images of Gr with spikes with some twisted Gr can be seen with aggregates which are very lightly covered with the matrix chains. The even more cloudy or diffused distribution over the entire surface of the composite material is evident in the case of the ArGrO system, providing a rougher surface in these composites. Since good dispersion and interaction of Grs with the matrix is essential for an improvement in mechanical properties, we find that most of the GrO sheets are well impregnated within the matrix showing very thick but diffused surfaces. These are distributed homogenously in these composites, showing that the chemical bonding in the ArGrO system had more influence on interfacial interaction than the multiple π–π interactions present in case of ArGr system.

### 3.5. Scanning Electron Microscopy (SEM)

**Figure 5:**
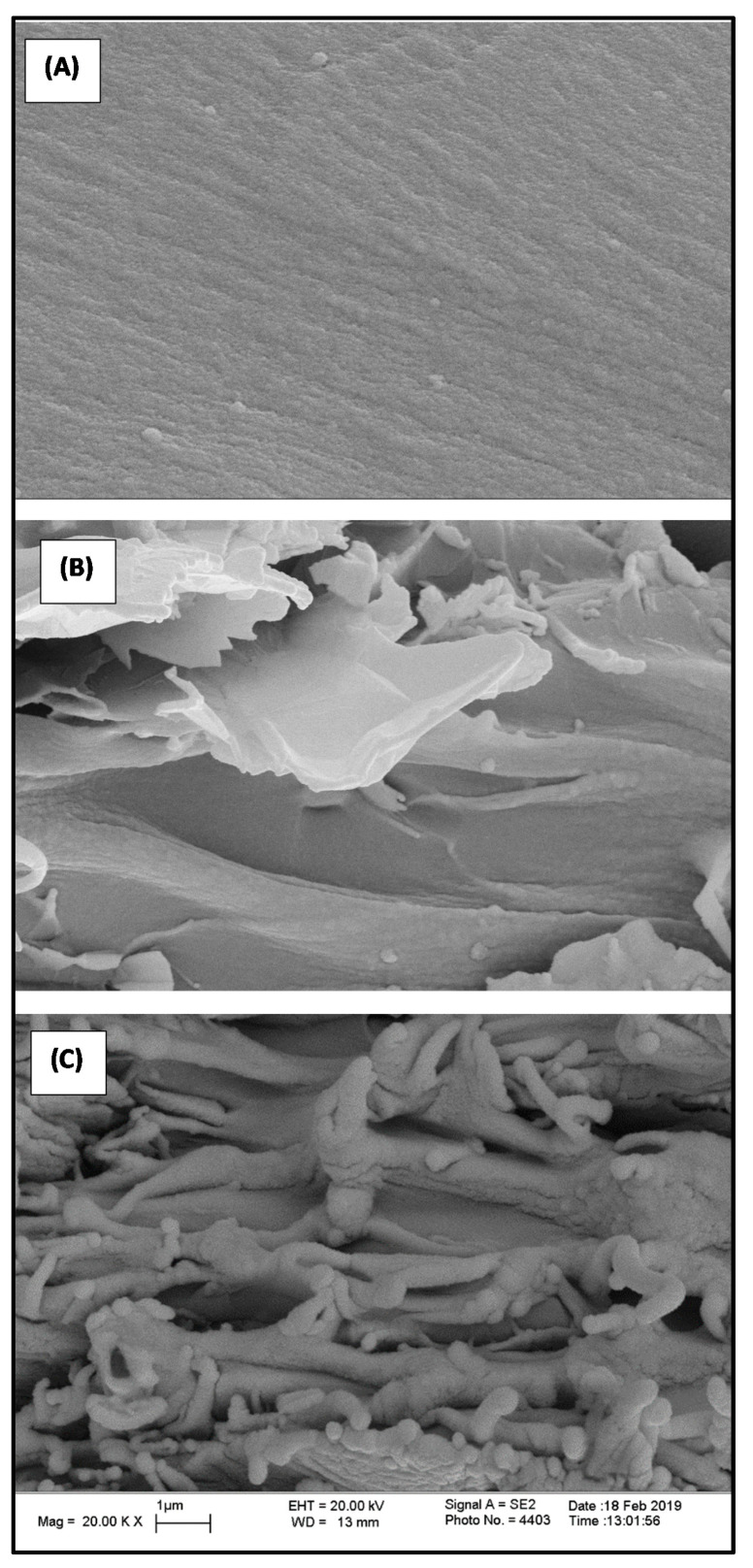
SEM micrographs of the fractured films: neat aramid (**A**); ArGr (**B**) and ArGrO (**C**) composites with 4 wt.% of graphene.

Morphologies of the neat matrix and that of ArGr and ArGrO composites with 4 wt % of graphene are shown in Figure 5A–C. The fractographs of neat aramid resin illustrate a relatively smooth surface with some stripes in the direction of fracturing force as shown in Figure 5A. On the other hand, in the ArGr film, the image of Gr in Figure 5B shows an increase in the thickness of plates, whereas the lateral size of sheets shows a marginal decrease. This is in agreement with the fact that Gr plates are less prone to lateral cleavage than oxidized ones. A remarkable increase in the thickness of graphene is seen in the ArGrO film, which is due to the presence of functional groups in the basal plane of GrO that have reacted with the polymer chains as is evident in Figure 5C. There is no sign of agglomeration of graphene sheets with each other. Roughness of the fractured surface of the composite is drastically increased on the addition of GrO sheets in the matrix in contrast to the ArGr composites. The irregular protuberances we find on the whole fractured the surface of the composite films due to the presence of GrO sheets, which are strongly bonded with matrix chains and were fractured without any de-bonding or pull-out of nano-layers. This is due to the covalent bond formation between the Ar matrix and GrO sheets. The improved dispersion, as seen in the micrograph (C), with the chemical bonding of graphene sheets with the matrix chains can have a profound effect on the ultimate mechanical properties of the composite material.

### 3.6. Dynamic Mechanical Thermal Analysis (DMTA)

**Figure 6:**
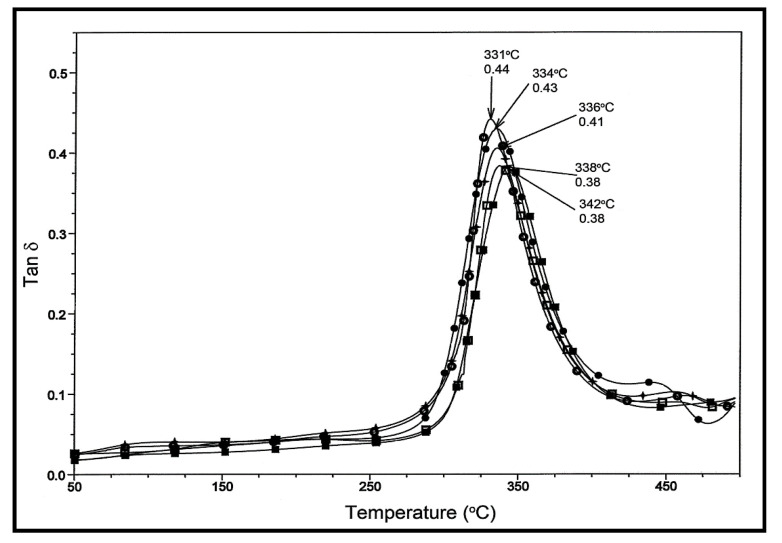

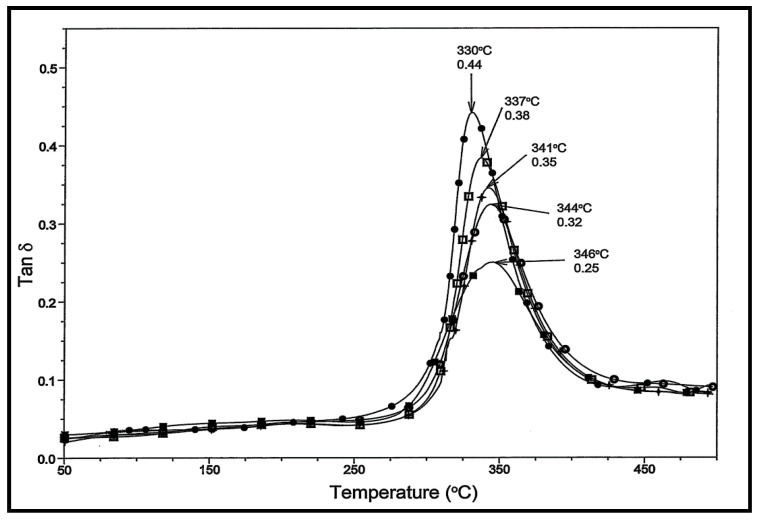
Temperature variation of tan δ in ArGr (upper) and ArGrO (lower) Composites; Gr Wt %: 0 (●), 2% (□), 4% (🟄), 6% (🞇) and 8% (■).

The DMTA was used to measure the visco-elastic properties of polymer materials under a controlled temperature and the same frequency conditions. Figure 6 shows the temperature variation of the tan δ for two types of composites with different loadings of Gr. The glass transition temperature (T_g_) involving alpha-relaxation in graphene-polymer composites was measured from the peaks of the tan δ curves plots against temperature. The T_g_ for the pure polymer was measured at 331 °C, which increased to 342 °C on the addition of pristine Gr, which indicates that the mobility of the polymer chains is restricted due to its interaction with the surface of Grs. This is further confirmed from the dampening effect on tan δ curves, which increases with the higher loadings of Gr in the matrix.

Liao et al. [64], in their extensive literature study from 62 research papers on the variation of T_g_ by inclusion of Grs in the polymer matrices, found that in the majority of cases the increase in the T_g_ was nearly 4 °C or more. Several of the studies showed no increase in T_g_, in particular when the melt blending or in-situ polymerization techniques were used. One of the reasons for this seems to be a poor dispersion due to high viscosity in such systems. It was suggested that in pristine Gr the conjugated C=C is chemically too stable to provide covalent bonding between graphene and the matrix polymer and thus the results is an almost unchanged T_g_ value in such composites. However, the nature of the polymer chains used as a matrix also matters a lot, as the pristine Gr by nature may not develop an interaction with the aliphatic type of polymer chain. In the pristine Gr, each carbon atom is linked with three sigma bonds to other carbon atoms but the pi electrons (π and π*), located above and below the sheets, are highly mobile. By using an appropriate polymer chain that has extended phenyl rings as carried out in the present work, the multiple π–π interactions can take place with exfoliated Grs and this secondary bonding with the matrix is responsible for the increase in the T_g_ in the present ArGr system.

The ArGrO composites in comparison to those using pristine Gr, however, have shown a stronger influence on the T_g_. The maximum value of T_g_ recorded was 348 °C. The larger damping in the tan δ curves showing more elastic behavior and an increase in T_g_ (Figure 6) observed for these composites is attributed to an improved adhesion with the matrix. The main reason for the large enhancement in T_g_ is the chemical bonding between GrO and the aramid chains. The wrinkled structures of GrO may also provide a stronger interlocking between the graphene and the matrix, thus increasing its elastic nature. Fu et al. [53] have reviewed the work done on graphene composites with polyamide-6. However, the matrix here discussed is concerned with aliphatic polyamides and the glass transition measured by DSC techniques was 188 °C which increased to 195 °C on the addition of graphene. Thermal stability of these composites was far below those given in the present work, mainly because of the resonance stabilization by the phenyl groups present on the chain and its interaction with graphene platelets.

**Figure 7:**
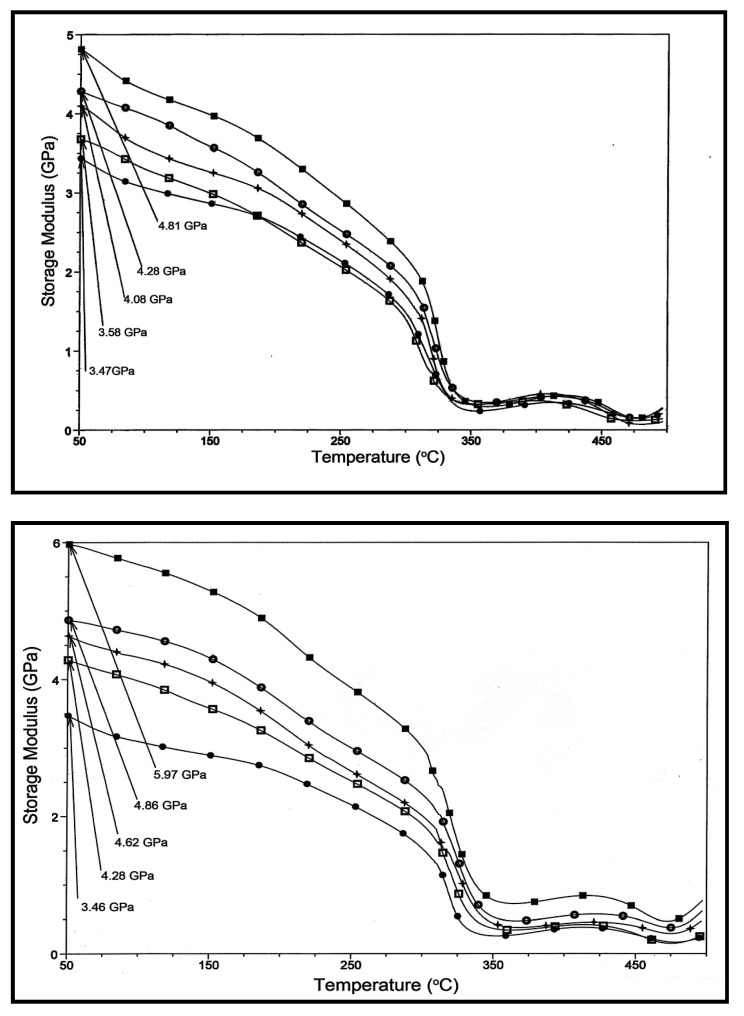
Temperature variation of Storage Modulus in ArGr (upper) and ArGrO composites; Gr Wt %: 0 (●), 2% (□), 4% (🟄), 6% (🞇) and 8% (■).

The variation of the storage modulus (E’) with temperature on Gr loading for the both types of composite systems is given in Figure 7. The glassy region modulus of the neat aramid at 50 °C is around 3.46 GPa, and with Grs loading it increases in both systems. The maximum value for the ArGr system was 4.81 GPa in comparison to the 5.97 GPa for the ArGrO system with 8 wt % loading.

The secondary or the primary bond interactions between the Grs and the aramid matrix help in providing a load-transfer mechanism which is responsible for improvement in the modulus values. The glassy state is more evident in case-bonded composites. With the rise in temperature, the modulus values decreased in the rubbery region for both the systems but it still remained higher in the neat matrix. Above 400 °C, the increase in the modulus values seems to be due to the cyclic stress induced during the measurements, which were taken in the tensile mode, thus causing some alignments of the polymer chains. A sharp drop in the E’ values witnessed above 480 °C is due to a softening of the polymer.

The modulus values for the ArGr system, as we enter the rubbery region with a rise in temperature, will fall sharply (see Figure 7) and come very close to the neat matrix in the temperature range (300–450 °C). This is because at a high temperature the secondary bond π–π interactions between the filler and the matrix become very weak and these interactions do not operate effectively as in the case of ArGrO where the chemical interactions still exist in the higher temperature range. In general we found a higher increase in modulus for the chemically bonded hybrids in comparison to the physically associated system.

**Figure 8:**
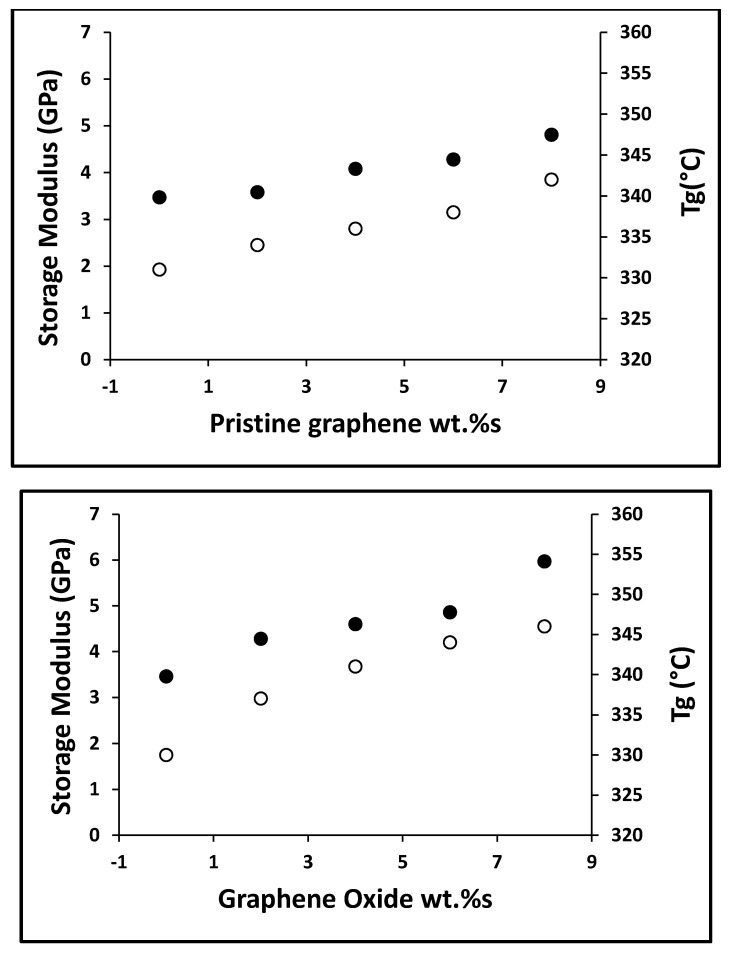
Variation of Glass transition Temperature (Tg) (ο) and the Storage Modulus (●) with Graphene contents in ArGr(upper) and ArGrO(lower) Composites.

The variation of the storage modulus in the glassy region and the Tg values for both types of composites are summarized in Figure 8. The large specific surface area of exfoliated Gr sheets induce secondary bond multiple π–π interactions with polymer chains and is responsible for increasing the thermal mechanical properties of the matrix. However, the influence of chemical bonding on the visco-elastic properties in the freezing of the motion of chain segments, i.e., local chain movements of the polymer chains, is much more. Our AFM and SEM studies (Figure 4 and Figure 5) confirm these findings as the surface of GRs was seen to be more impregnated and modified with the polymer chains in ArGrO composites.

### 3.7. Tensile Properties

**Figure 9:**
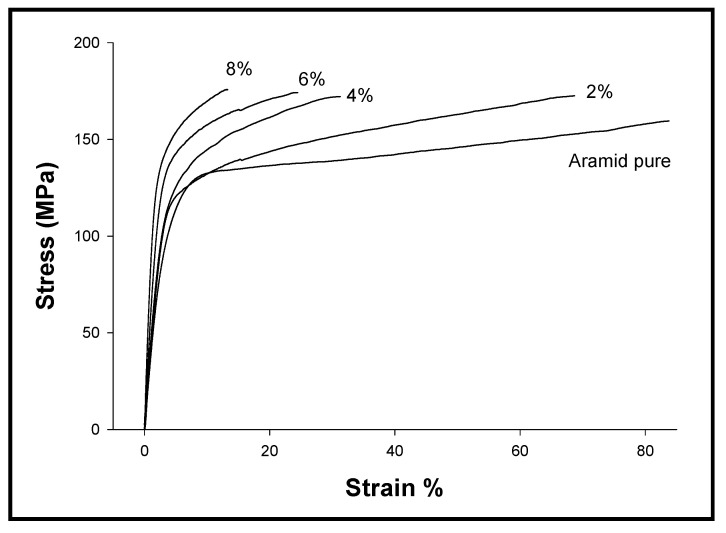
Stress-strain curves for ArGr composites with different Wt.% of Gr.

**Figure 10:**
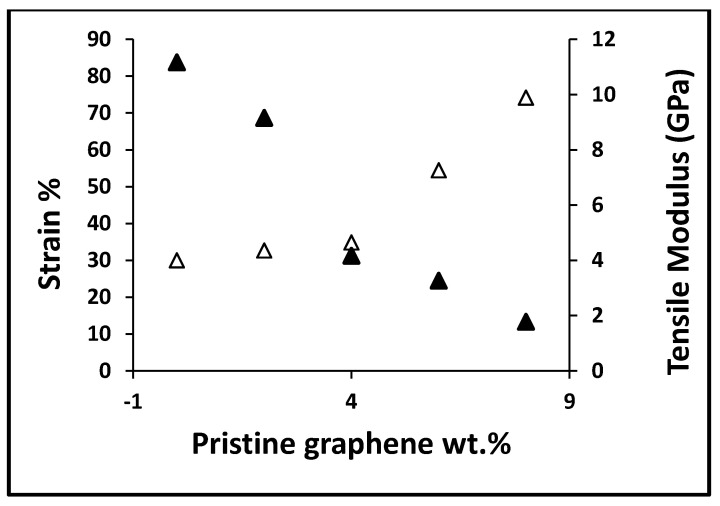
Tensile Modulus (GPa) (∆) and Strain variation (▲) in ArGr composites with different wt.% of Gr.

**Figure 11:**
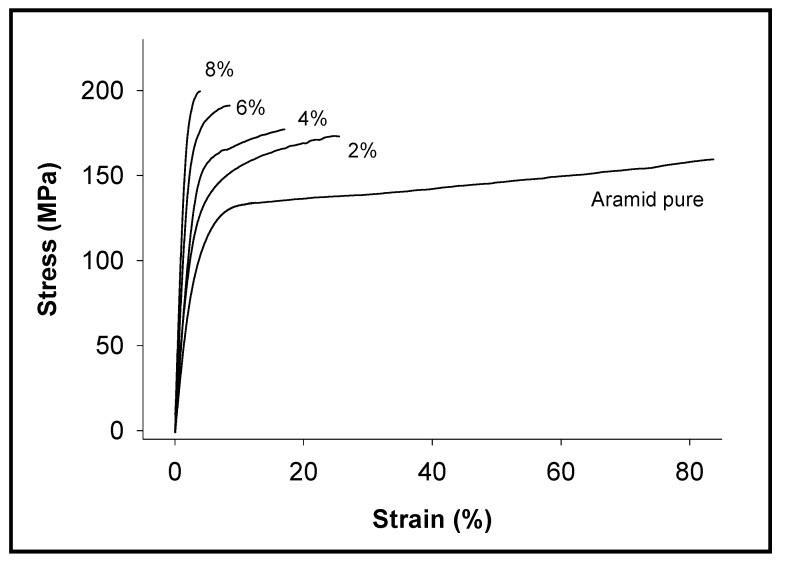
Stress-strain curves for the ArGrO composites with different Wt.% of GrO.

The stress-strain curves and the ultimate tensile strength of the composites were obtained according to ASTM D-882. The data obtained have been given in Table 1. The typical stress strain-curve for the ArGr system has been shown in Figure 9. As seen in this Figure, the tensile strength for the neat polymer is 159.11 MPa and with the addition of nano-sheets it has increased; the maximum stress at the break point observed is 176.50 MPa (Figure 10). From the slope of the initial elastic part of the stress-strain curve, values of Young’s modulus were calculated. Young’s modulus for the pure polymer was 4.00 GPa, which increased on the addition of Gr significantly and a maximum value of 9.89 GPa. This is mainly due to the high strength of the graphene sheets helping the matrix material to bear an external force, dispersion pressure and load. A higher increase in the Young modulus and tensile strength was observed in the case of the ArGrO-bonded system (Figure 11).

**Figure 12:**
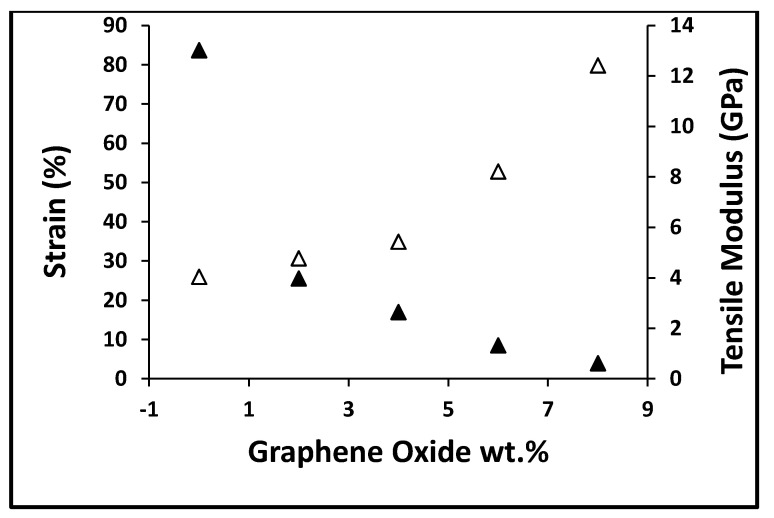
Tensile Modulus (GPa) (∆) and Strain variation (▲) in ArGrO composites with different Wt.% of GrO.

The maximum stress at the break point was 201.14 MPa and the modulus value of 12.43 GPa was observed in the case of the chemically bonded system (Figure 12). The strain rate decreased as the filler content increased in both cases. In the conventional composites, interphase is mainly considered to contribute to the load transfer effect. The large surface to volume ratio in GRs and its chemical bonding and the magnitude of interphase developed in the matrix is substantial as observed in the AFM and SEM results. The higher increase is due to strong chemical bonding between the phases, whereas in a physically bonded system, the slippage of the polymer chain at the interface is more probable under the tensile stress due to weak interfacial forces. The functional groups on GrO and their chemical reaction help the interfacial load transfer via GrO/polymer chain bonding, thus leading to an improvement in mechanical strength. Such promising lightweight materials with a tensile strength around 200 MPa can be considered for ultra-strong membranes/coatings in the high tech industry.

### 3.8. Thermogravimetric Analysis (TGA)

**Figure 13:**
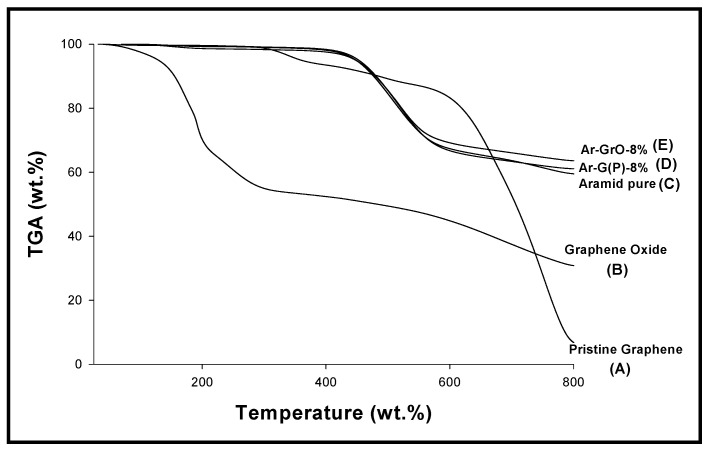
TGA Thermograms for Gr, GrO, Ar neat, ArGr and ArGrO films with 8 Wt.% Graphene.

**Figure 14:**
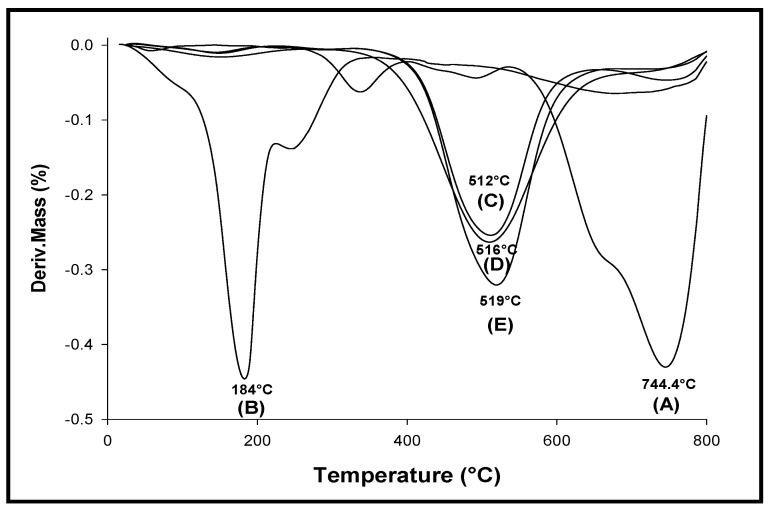
First derv. of thermograms showing thermal decomposition temperature for Gr(A), GrO(B), Ar neat(C) and ArGr (D) and ArGrO (E) both with 8 wt.% graphene.

Thermogravimetric analysis of Gr, GrO, aramid neat resin and that of the ArGrO composite with 8 Wt % of GrO is depicted in Figure 13. The first derivative of thermograms was also plotted against temperature, showing clearly the peak of the thermal decomposition temperature in each case (Figure 14).

Pristine Gr under an inert atmosphere is thermally stable (A). Its weight dropped slightly around 340 °C, which may be due to a detachment of adsorbed oxygen atoms and there is almost no mass loss in Gr before 700 °C.

In the case of GrO, the mass loss occurred much earlier, i.e., from 150 to 300 °C. The GrO contains various oxygen functional groups; i.e., hydroxyl, epoxy, carbonyl and carboxylic acid groups, and these functional groups can be decomposed earlier by heat treatment (B). The decomposition of labile oxygen functional groups such as carboxylic, lactone and anhydride, can take place within 400 °C but the more stable functional groups like phenol and carbonyl may decompose at a higher temperature.

The aramid chains are also very stable thermally (C) due to their aromatic character of the backbone. The Kevlar© chain has an excellent thermal stability and decomposes at around 528 °C, whereas Nomex© has a lower thermal decomposition temperature at around 428 °C [65]. The copolymer of these in the present case showed a thermal decomposition temperature (C) at 512 °C (Figure 14). For the ArGr and Ar GrO composites, thermal decomposition temperatures (shown as D and E) are observed at 516 °C and 519 °C respectively and have shown only a slight increase. The network of chemically bonded GrO in the matrix can protect to some extent the aramid matrix by decreasing the mobility of free radicals produced during the degradation process. In contrast to Gr, the GrO in the polymer composite can capture the free radicals generated during thermal decomposition but as aramid chain itself is highly stable there cannot be so much of an increase in the decomposition temperature of composites as observed in the case of graphene composites with an aliphatic type of polymer chain.

## 4. Conclusions

For high performance nanocomposite aromatic polyamides, e.g., Kevlar or Nomex, having repeated phenyl groups in the chain can be considered the best candidate as a matrix due to the advantage of π–π stacking with Gr fillers. But these polymers are insoluble in the organic solvents. By a careful choice of matrix like the Kevlar–Nomex copolymer, it is possible to make their composites by solution blending with Gr fillers. Though physical bonding with the conjugated surface of the pristine Gr through π-π stacking is possible, the interfacial interactions can be further increased by developing chemical bonding between GrO and the functionalized polymer chains. The chemically bonded composites in the present study have been found to possess a higher magnitude of interphase as observed in our AFM and SEM studies. The resulting composites possessed higher mechanical properties and the glass transition temperature in comparison to the physically associated composites using pristine Gr.

## Figures and Tables

**Table 1:** Tensile Properties of Ar-Gr and Ar-GrO Composites.

Sample with Gr-Wt.%	Tensile Modulus (GPa)		Tensile Strength (MPa)		Strain at Break (%)	
	Ar-Gr	Ar-GrO	Ar-Gr	Ar-GrO	Ar-G	Ar-GrO
Ar neat	4.00	4.00	159.51	159.51	83.78	83.78
Ar-Gr-2%	4.39	4.77	172.59	174.27	69.92	25.66
Ar-Gr-4%	4.62	5.44	172.75	177.24	32.23	17.02
Ar-Gr-6%	7.26	8.21	174.19	193.22	24.56	8.84
Ar-Gr-8%	9.89	12.43	176.50	201.14	13.35	3.99
